# Light-Powered Liquid Crystal Polymer Network Actuator Using TiO_2_ Nanoparticles as an Inorganic Ultraviolet-Light Absorber

**DOI:** 10.1021/acsomega.3c00417

**Published:** 2023-03-10

**Authors:** Zhila Alipanah, Mohammad Sadegh Zakerhamidi, Hossein Movla, Batool Azizi, Igor Muševič, Amid Ranjkesh

**Affiliations:** † Faculty of Physics, University of Tabriz, Tabriz 5166614761, Iran; ‡ Photonics Center of Excellence, University of Tabriz, Tabriz 5166614761, Iran; § Central laboratory, University of Tabriz, Tabriz 5166614761, Iran; ∥ Condensed Matter Department, J. Stefan Institute, Jamova 39, Ljubljana 1000, Slovenia

## Abstract

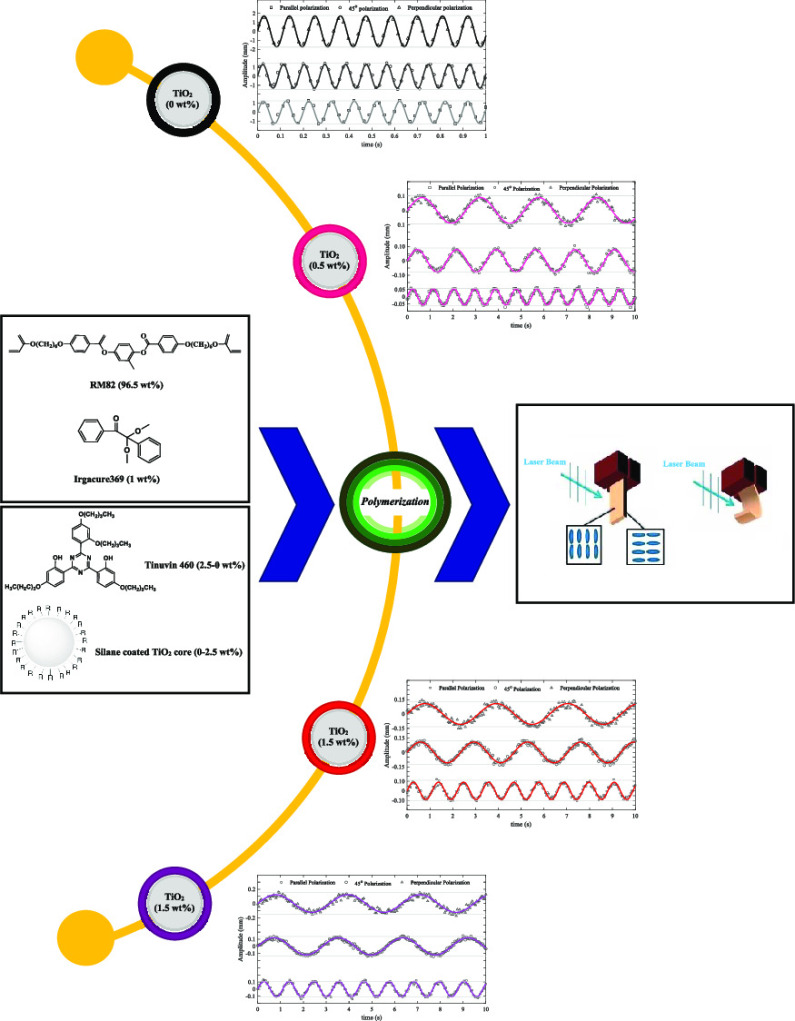

Recently, the design and fabrication of light-powered actuators have attracted immense attention because of the manufacturing of intelligent soft robots and innovative self-regulating devices. Accordingly, a liquid crystal polymer network (LCN) provides a promising platform due to its reversible and multistimulus-responsive shape-changing behaviors. In particular, doping nanoparticles with exclusive properties into the LCN can produce interesting results. In this work, we investigated a TiO_2_ nanoparticle-based LCN polymer light-powered actuator. TiO_2_ nanoparticles as an inorganic ultraviolet (UV)-light absorber can substantially affect the LCN polymer’s oscillatory behavior. Our results demonstrate that the oscillation characteristics are directly influenced by the presence of nanoparticles, and we studied the influencing factors. The effectiveness of the elastic modulus, thermomechanical force, and curvature was investigated using different weight percentages of TiO_2_ nanoparticles. Our results show that, in the presence of TiO_2_ nanoparticles, the polymer chain order and inter-chain interactions in the polymer matrix as well as the structural deformation of relevant polymer surfaces are changed.

## 1 Introduction

In today’s world, movement has penetrated everywhere in human life, which serves as the inspiration for modern industries.^1−3^ Indeed, the oscillating movement that is present in all of nature, such as leaves swinging with the breeze, birds flapping their wings, ocean waves, and heartbeat, has attracted scientists’ attention.^2,4,5^ In addition, oscillatory behaviors can be employed in practical applications such as soft robots and self-cleaning surfaces.^6−8^ Due to this appeal, many efforts have been devoted to designing and manufacturing self-oscillating actuators with self-determining movement in recent years.^4−9^ For this reason, crucial features such as enabling wireless, spatial, and temporal control should be given attention. Light can play a unique role in features of light-responsive soft actuators because it can be highly controllable in an untethered way with spatial and temporal accuracy.^9,10^

For this reason, the use of light-sensitive materials is aimed in this work. Among different aspects of selecting the suitable material, the degree of flexibility and the capacity to change shape with a reasonable response time should be considered.^11,12^ These features help us to overcome rigid actuator issues such as finite freedom and relatively complex control systems.^13^ The liquid crystal polymer network (LCN), which combines the mechanical capabilities of polymers with the anisotropic nature of LC, has been prioritized as a candidate among the materials employed in this discipline. The LCN can produce controlled oscillating motions, and these materials have recently gained popularity for employment in mobile components. Moreover, they have sufficient elasticity and the capacity to alter shapes against light irradiation.^14−16^

Recently, research in this field has been focused on increasing the scope of these light-powered actuator applications. However, the integration of the exclusive properties of the LCN polymer with the appropriate dopant can provide the possibility of obtaining valuable results.^17−22^ Ultraviolet (UV)-light absorbers are one of the best candidates among the dopants. The UV-light absorbers are a great selection and protect the actuator against UV-light radiation having a high energy of UV light and potentially breaking of intra- and intermolecular bonds and deteriorating the physical and chemical characteristics of the LCN polymer.^26^ For this reason, several research studies have used organic UV-light absorbers, which have achieved self-sustained oscillation along with the improvement of the photothermal effect.^23−25^ Due to their superior performance to photo-chemical types in quick actuation and highly reversible responses with just one light wavelength, the emphasis is placed on photothermal effect-based actuators to accomplish this goal.^27^

Inorganic UV-light absorbers are chosen because of their nontoxicity and high chemical stability.^28^ One excellent choice among them is titanium dioxide (TiO_2_), a chemically inert semiconducting substance that does not affect the nature of the host matrix.^29^ Due to this substance’s special characteristics, including its high refractive index, non-flammability, and high thermal capacity, a wide range of applications have been assigned to it, including wood coatings, solar cells, water purification systems, and cosmetics.^30−33^ Integrating the TiO_2_ nanoparticles’ exclusive properties with the LCN polymer’s capabilities can produce exciting outcomes.

In this work, a TiO_2_-doped light-powered LCN polymer actuator was fabricated to explore the impact of stated nanoparticles on the oscillatory behavior. The oscillation characteristics under the influence of the weight percentage of nanoparticles were investigated as well. Moreover, investigations were done on different radiant polarizations, the effect of nanoparticles on chemical bonds in the polymer matrix using FT-IR spectroscopy, the absorbance, molecular orientations with SEM images, and the surface morphology by AFM techniques. Indeed, by studying TiO_2_ nanoparticles on the resultant light-induced oscillatory behavior, we can get helpful information about the LCN polymer’s elastic modulus, thermomechanical force, and curvature radius.

## 2 Experimental Section

### 2.1 Materials

RM82 (*M*_w_ = 120,000, Sigma Aldrich) and Irgacure369 (provided by Ciba, Basel, Switzerland) were used as the nematic reactive mesogen and photoinitiator, respectively. Also, silane-coated rutile TiO2 nanoparticles (SSNANO Co., Ltd.) with an average size of 20 nm were selected as an inorganic UV-light absorber. Meanwhile, to carry out the studies as accurately as possible, the TiO2 nanoparticles were mixed with Tinuvin460 (BASF) as an organic UV-light absorber, which has already been studied^25^ in specific weight percentages as listed in Table. 1. These materials cause a low glass transition temperature (*T*_g_) in the respective LCN polymer films.^23,24^ It is also noteworthy that, further, polyimide X610-33C (JNC Corporation, Tokyo, Japan) and polyimide SE-5662 (Nissan) were employed to create surface alignment layers with planar and homeotropic molecular orientations, respectively, to achieve a splay molecular orientation in the LCN polymer matrix.

**Table 1:** Specified Weight Percentage (wt %) of Tinuvin 460 and TiO_2_ Nanoparticles to Polymerization of the LCN Polymer within the Polymerized Films’ Density

LCN polymer	Tinuvin 460 (wt %)	TiO_2_ nanoparticle (wt %)	density (kg/m^3^)
LCN	2.5	0	1220
LCN/C-I	2.4	0.1	1223.1
LCN/C-II	2.2	0.3	1229.3
LCN/C-III	2	0.5	1235.5
LCN/C-IV	1.8	0.7	1241.7
LCN/C-V	1.5	1	1251
LCN/C-VI	1	1.5	1266.5
LCN/C-VII	0.5	2	1282
LCN/C-VIII	0	2.5	1297.5

### 2.2 Polymerization

The photo-polymerization method was carried out for nine mixtures of the stated LCN with same weight percentages of RM82 and Irgacure369 (96.5 and 1 wt %, respectively). The weight percentages of Tinuvin460 and TiO_2_ nanoparticles are variable, as listed in Table 1, which are adjusted so that their sum equals 2.5 wt %. With the systematic increase in the weight percentage of TiO_2_ nanoparticles, it is possible to examine their impact on the desired photoresponsive behavior more precisely. All mixtures were dissolved in dichloromethane (Merck Co., Ltd) with the highest available purity. After ensuring the homogeneous mixing of nanoparticles and complete evaporation of dichloromethane using a Laboratory Mixer (50 rpm, 30 min, 50 °C), the LC cells were filled with the remaining mixtures. In this way, the quartz sheets were coated using the mentioned polyimides through the spin-coating technique with the specified program: Program 1: 1200 rpm for 10 s; Program 2: 2000 rpm for 50 s. Then, the coated sheets were prebaked at 180 °C for 30 min. After finishing the coating step, to improve the orientation, microgrooves are created on the planar alignment layer by rubbing on velvet cloth. Subsequently, after gluing planar and homeotropic alignment sheets with UV glue with a set gap of 20 μm, the prepared cells are filled with the respective mixtures in the isotropic phase (110 °C) using capillarity. To complete the polymerization process, the mixture samples were exposed to a 365 UV light (power density 10 mW/cm^2^) for 35 min at the nematic phase of mixtures (92 °C). Finally, post curing was done at 135 °C for 15 min.

## 3 Characterization

### 3.1 Characterization Instruments

To study the physical and chemical characteristics of the LCN polymer and examine the impact of utilized TiO_2_ nanoparticles on the related polymer networks’ chemical bonds, a Vertex 70 FT-IR spectrophotometer spanning the wavenumber range 400–4000 cm^–1^ was used. Additionally, checking the molecular orientation in the polymer bulks is done by examining the images recorded by scanning electron microscopy (MIRA3 FEG-SEM). In addition, this microscope allows for checking the dispersion of nanoparticles in the polymer matrix by an energy dispersive X-ray mapping (EDX mapping) technique. To check the impact of utilized nanoparticles on the LCN polymer’s morphology, a Nanosurf Mobile S device was used to conduct an atomic force microscopy (AFM) study. To investigate the utilized nanoparticles’ role on light absorbance, a double-beam Shimadzu UV-2450 UV–visible spectrophotometer spanning a wavelength range of 200–900 nm was employed for obtaining the UV–vis absorption spectra of samples. To determine the elastic modulus, an ASTM/D638/Shimadzu testing machine was used. To record the temperature of the LCN polymer during oscillation, a Xenics Gobi thermal camera was used in this work.

### 3.2 Adjusted Experimental Arrangement

**Figure 1:**
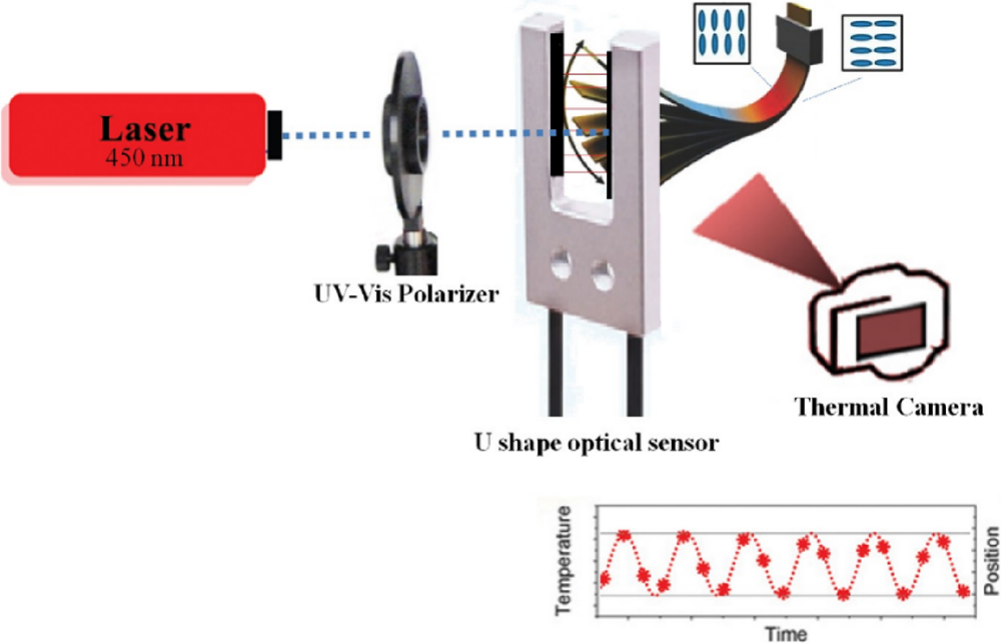
Experimental arrangement to achieve the light-induced oscillating motion in the LCN films.

To obtain the light-induced oscillating motion, polymerized LCN films are placed in the experimental setup, as shown in Figure 1. It is very important to eliminate the factors causing disturbances in the oscillatory behavior. Because of this, we can investigate the impact of the utilized TiO_2_ nanoparticles on the LCN behavior as precisely as possible. This was accomplished by selecting a radiant light source with a wavelength of 450 nm, which is outside the maximum absorption bands of TiO_2_ and Tinuvin 460 (see Figure S1a,b). Further, a Fresnel rhomb (Newport) polarizer was used to adjust the light polarization direction. In this regard, the polymerized LCN films are all cut in a rectangular shape in the dimension of 2 mm × 1.8 cm so that the generated grooves run parallel to the cantilever’s long-axis direction. Then, the sliced films are held in place by a clamp as the planar alignment surface is irradiated by the light source. In addition, the uniformity of the studied LCN films’ dimensions was checked using an optical microscope and scaled slides with an accuracy of 0.01 mm. Further, the mass of these films was measured by a Sartorius MC 5 scale with a capacity resolution of 5.1 g × 1 μg. Subsequently, the density of the LCN film was calculated by the dimension and mass of all films. The obtained values are listed in Table 1. A 120 Hz frame rate video camera was used to record the resultant light-induced oscillating motions. Finally, using video analysis software (Tracker), the required parameters of the resultant oscillations were extracted from the collected images.

## 4 Results and Discussion

### 4.1 Chemical and Physical Characteristics

To precisely analyze the LCN polymer’s light-induced oscillatory behavior and controlling factors, the disturbance-causing factors should be considered. Thus, the following investigations were carried out on the physical and chemical characteristics of polymerized LCN films.

**Figure 2:**
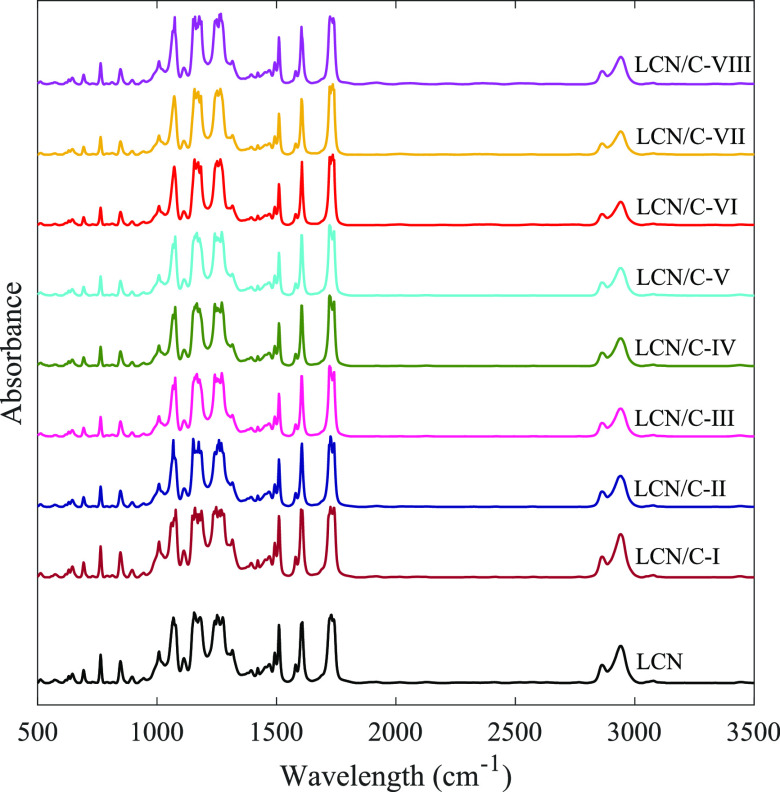
FT-IR spectra of all LCN films.

First, the impact of TiO_2_ nanoparticles on the chemical nature of the LCN polymer was studied. To investigate this feature, the FT-IR spectra of all LCN films were recorded, and the spectrum associated with the TiO_2_-doped LCN films was compared to those related to non-doped LCN films. As shown in Figure 2, the FT-IR spectra of all films show the same trend. Additionally, neither a new IR bond nor a change in the wavelength of the IR-active functional groups was observed. This indicates the failure of chemical bonds between the utilized nanoparticles and the LCN polymer matrix.^34^ This finding allows for the most precise execution of the appropriate research by guaranteeing the neutrality of the utilized TiO2 nanoparticles.

**Figure 3:**
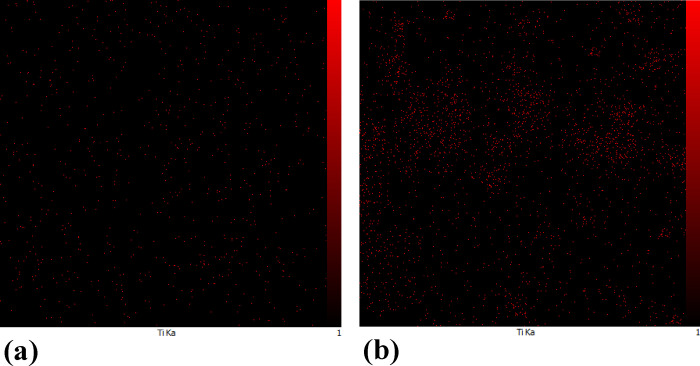
EDX map from the dispersion of Ti in the number of LCN films: (a) LCN/C-III; (b) LCN/C-VIII.

To consider the significance of the nanoparticle dispersion in the polymer matrix on its photoresponsive behavior,^35,36^ the dispersion of the utilized nanoparticles was analyzed using the EDX-mapping technique. The dispersion of titanium (Ti) in the corresponding polymer matrices was examined for this goal. The related maps are shown in Figure 3, which demonstrates that the specified element is homogeneously distributed throughout the considered LCN polymer matrices. This presumes that the related dispersion in the polymer matrix with the highest weight percentage of TiO_2_ nanoparticles (2.5 wt %) acquires a disturbance. This suggests that, when using significant weight percentages of nanoparticles, the aggregation will manifest itself. The pertinent findings in turn confirm the effectiveness of the photothermal effect in the considered polymer matrices from the dispersion of the utilized TiO_2_ nanoparticles. Additional related images are provided in Figure S2.

**Figure 4:**
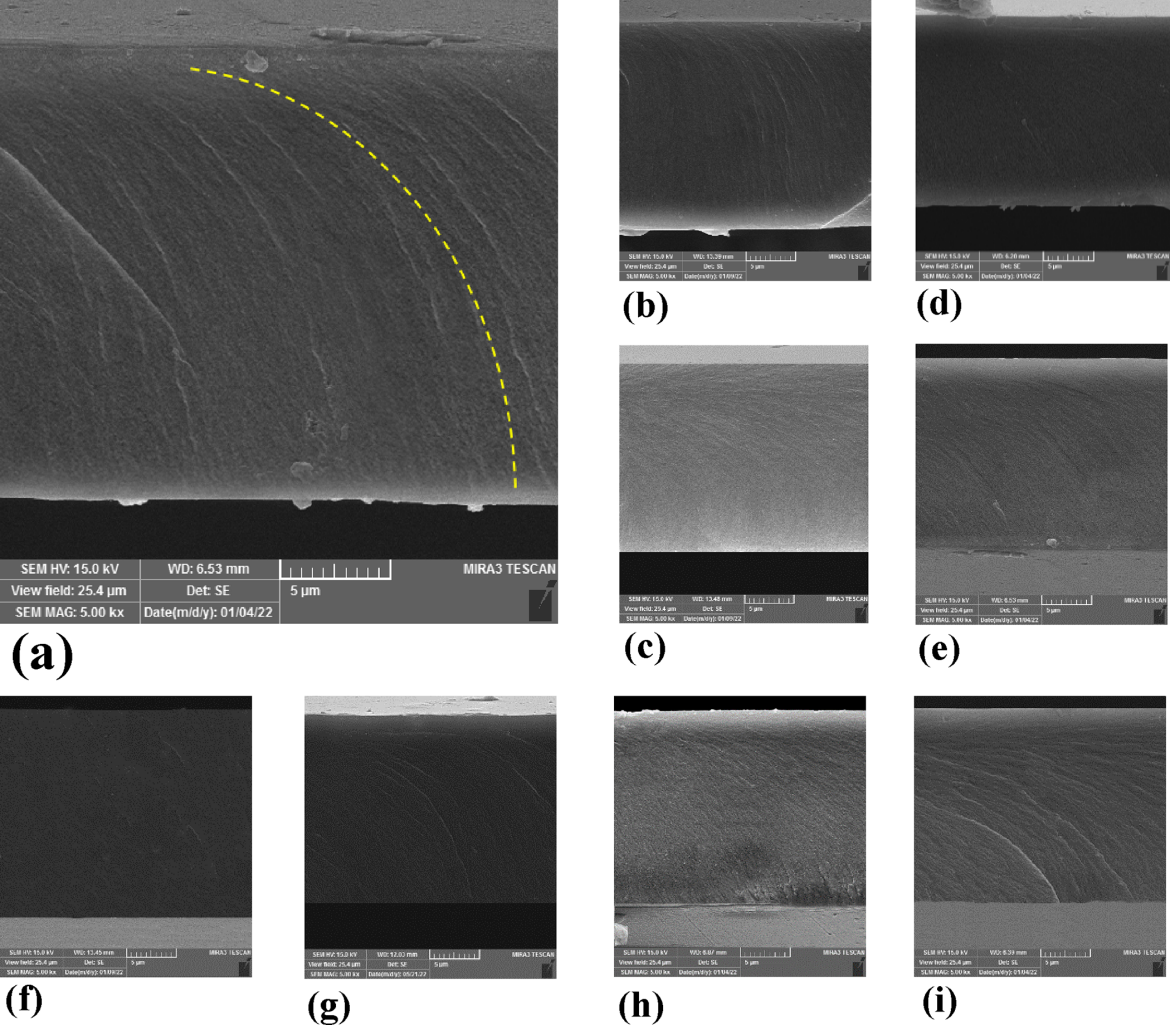
SEM images of all LCN films’ cross section: (a) LCN, (b) LCN/C-I, (c) LCN/C-II, (d) LCN/C-III, (e) LCN/C-IV, (f) LCN/C-V, (g) LCN/C-VI, (h) LCN/C-VII, and (i) LCN/C-VIII. The top and bottom surfaces are planar and homeotropically oriented, respectively.

It should be noted that the morphology is another influencing factor on the photoresponsive behavior of the LCN polymer.^25,37^ SEM and AFM images of all LCN films’ cross-sections and surfaces, respectively, were captured for this purpose. The analysis of images offers the chance to look into the influence of the utilized nanoparticles on the splay molecular orientation in the considered polymer bulks. Apart from the molecular orientation’s impact on the LCN polymer’s photoresponsive behavior, the accomplishment of thermal anisotropy in the splay structures plays a significant role in the formation of photo-induced oscillating motion.^38^ By contrasting the SEM images of the TiO_2_-doped LCN films and the non-doped one, it is conceivable to identify the presence of the desired splay molecular homeotropic molecular orientation as present in all images. This finding in turn indicates the orientation in the considered polymer bulks (see Figure 4). The transfer from a planar to the relevant molecular orientation is unaffected by the utilized nanoparticles.

**Figure 5:**
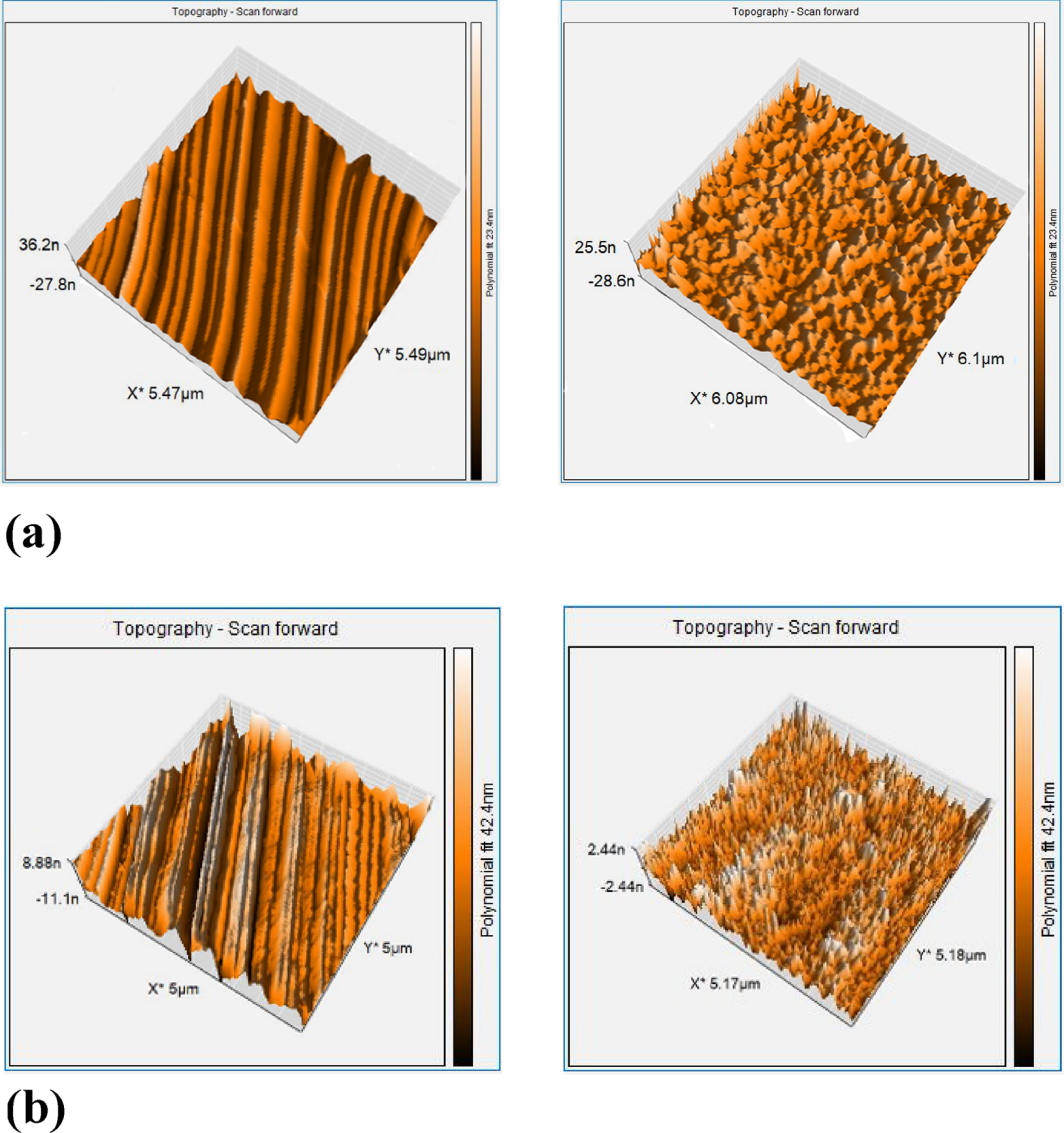
AFM images of planar (images on the left) and homeotropic (images on the right)-oriented surfaces: (a) LCN (b) LCN/C-VIII.

Subsequently, AFM images of all polymer surfaces were captured, including both planar and homeotropic molecular orientations. The images of non-doped LCN and LCN/C-VIII are given in Figure 5, and the images related to the rest of the TiO_2_-doped films are presented in Figure S3. In this regard, the non-uniformity of the respective polymer surfaces’ roughness is revealed by comparing the related roughness of the TiO_2_-doped LCN films with the non-doped LCN film. Even though the planar and homeotropic-oriented surface roughness (*S*_a∥_ and *S*_a⊥)_ are not the same in all of them, the surface roughness in the TiO_2_-doped films has decreased in comparison to the non-doped LCN. This result expresses the influence of TiO_2_ nanoparticles on the LCN chain structural tension of relevant polymer surfaces. Because the molecular orientations are not the same on the side surfaces of the considered LCN films, the surface roughness ratio (*S*_a⊥_/*S*_a∥_) was calculated in order to provide the possibility of investigating accurately. The values listed in Table 2 show that the specified ratio decreases when the utilized nanoparticles are present, while the surface roughness ratio of TiO_2_-doped films rises as the weight percentage of nanoparticles increases. In this manner, it appears that an increase in the pertinent weight percentage has a more significant impact on the structural chain tension in the planar-oriented LCN layer than in the homeotropic-oriented one.

**Table 2:** Surface Roughness Ratio (*S*_a⊥_/*S*_a∥_) and Absorption Coefficient (β) at the Radiation Wavelength (450 nm) of the LCN Films

polymer film	*S*_a⊥_/*S*_a∥_	β (1/cm)
LCN	1.88	39.61
LCN/C-I	1.34	26.41
LCN/C-II	1.36	25.15
LCN/C-III	1.39	17.45
LCN/C-IV	1.41	14.07
LCN/C-V	1.46	8.12
LCN/C-VI	1.51	7.92
LCN/C-VII	1.57	7.31
LCN/C-VIII	1.65	5.18

**Figure 6:**
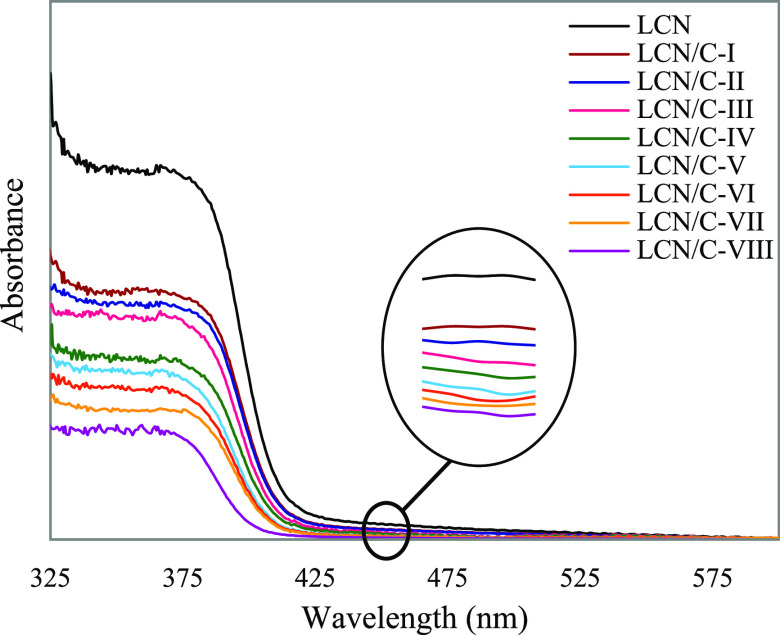
UV–vis absorption spectra of all LCN films.

However, it should be noted that the amount of irradiation light absorption by the LCN polymer is another factor affecting its oscillatory behavior because the light absorbance influences the effectiveness of the photothermal effect, which controls the corresponding oscillatory behavior. Therefore, the absorption spectra of all the LCN films were recorded as shown in Figure 6. For this purpose, the relevant spectra at the 450 nm wavelength, which is the light wavelength, were investigated. Next, to investigate as precisely as possible, the absorption coefficient (β) of all films in this wavelength was determined through Beer–Lambert’s law.^39^ As listed in Table 2, the results demonstrate a decrease in the related coefficient in the presence of TiO_2_ nanoparticles. It is noteworthy that the values calculated for the LCN films are the same in all the considered polarizations to investigate the oscillatory behavior separately. Additionally, in the TiO_2_-doped films, the absorption coefficient drops as the weight percentage of nanoparticles rises. Regarding the operation of TiO_2_ as a UV-light protector, the resultant declining tendency can confirm the increased light scattering in the LCN films with a higher percentage of the TiO_2_ dopant.

### 4.2 Oscillation Characteristics

To achieve the light-induced oscillating motion, all films were arranged in the experimental setup, as shown in Figure 1. Then, we studied the influencing factors on the oscillatory behavior of LCN films based on their chemical and physical characteristics. For this purpose, all films were attached to the clip so that the place of light radiation was approximately 2 mm lower than the attachment point, which is known as the hinge point. In this regard, all the films started to oscillate after bending toward the light source. Every obtained oscillation persisted without interruption, and the oscillatory behavior of all the films was recorded in the same way for 60 min by a 120 Hz frame video camera incorporated into the experimental arrangement to explore their characteristics as precisely as possible. To progress the relevant investigations, all films were exposed to radiant light with polarizations of 0 (parallel), 45, and 90° (perpendicular). The corresponding polarizations are established according to the orientation of the cantilever’s long axis concerning the radiant light source. The existence of structural anisotropy in the corresponding LCN network, which is the result of the presence of a splay molecular orientation, in turn causes the non-uniformity of the LCN polymer’s oscillatory behavior with different polarizations of radiant light.^40^

**Figure 7:**
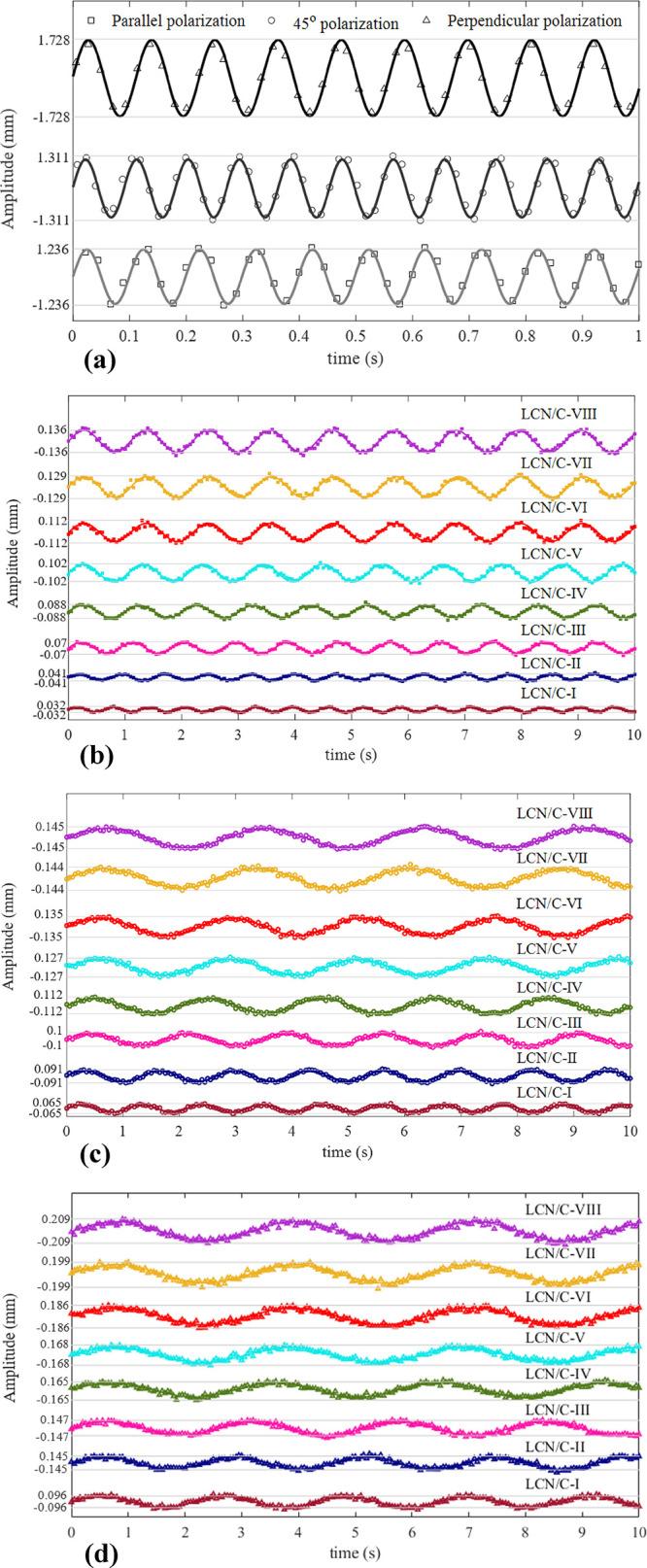
Oscillation pattern with irradiation of polarized light: (a) LCN with all polarizations, (b) parallel polarization, (c) 45° polarization, and (d) perpendicular polarization. Temperature changes between the LCN films’ surfaces with increasing the weight percentage of TiO_2_ nanoparticles: 1–0.5 °C.

For advancing the relevant investigations, by reviewing the recorded videos, all oscillation characteristics were extracted by Tracker software and then determined by the sinusoidal fitting procedure. The findings of the proper examinations revealed that, during the recording period, all oscillations were consistently stable in a consistent amplitude and frequency and continued without any flaws. The pattern of resulting oscillations for 10 s equally is given in Figure 7, and the relevant characteristics are listed in Table 3. Notably, the non-doped LCN’s oscillation frequency is high compared to the TiO_2_-doped types; to show the corresponding oscillation pattern as clearly as possible, its pattern is drawn for 1 s. Additional related oscillation patterns are provided in Figure S4a–h). However, it should be noted that the temperatures of all films during oscillation are measured by a Xenics Gobi thermal camera with a 0.01 °C thermal resolution. The results showed that there is a temperature difference between planar and homeotropic-oriented surfaces of all films. In addition, there is a non-uniformity in the temperature between the different irradiation light polarizations where the difference between temperatures is roughly 2–1.5 °C in the LCN. In the TiO_2_-doped films, this temperature difference decreases with increasing the doping weight percentage. However, due to having a high absorbance of TiO_2_ at the 450 nm wavelength compared to Tinuvin 460 (see Figure S1), the high heat capacity of TiO_2_ causes a variation trend. Thus, by increasing the doping weight percentage, the temperature difference between the two oriented surfaces of the studied films decreases to 0.5 °C.

**Table 3:** Oscillation Frequency (*f*) and Amplitude (*A*) during Irradiation with Polarized Light, and Also Their Frequency Rate (*f*_⊥_/*f*_∥_)

	frequency (Hz)			amplitude (mm)			
	0° (parallel)	45°	90° (perpendicular)	0° (parallel)	45°	90° (perpendicular)	*f*_⊥_/*f*_∥_
LCN	10.04	11.023	8.95	1.236	1.311	1.728	0.891
LCN/C-I	1.58	0.935	0.465	0.032	0.065	0.096	0.294
LCN/C-II	1.319	0.755	0.433	0.041	0.091	0.145	0.328
LCN/C-III	1.11	0.577	0.382	0.07	0.1	0.147	0.344
LCN/C-IV	0.997	0.497	0.346	0.088	0.112	0.165	0.347
LCN/C-V	0.937	0.435	0.326	0.102	0.127	0.168	0.348
LCN/C-VI	0.911	0.427	0.319	0.112	0.135	0.186	0.350
LCN/C-VII	0.908	0.369	0.32	0.129	0.144	0.199	0.352
LCN/C-VIII	0.901	0.323	0.318	0.136	0.145	0.209	0.353

**Figure 8:**
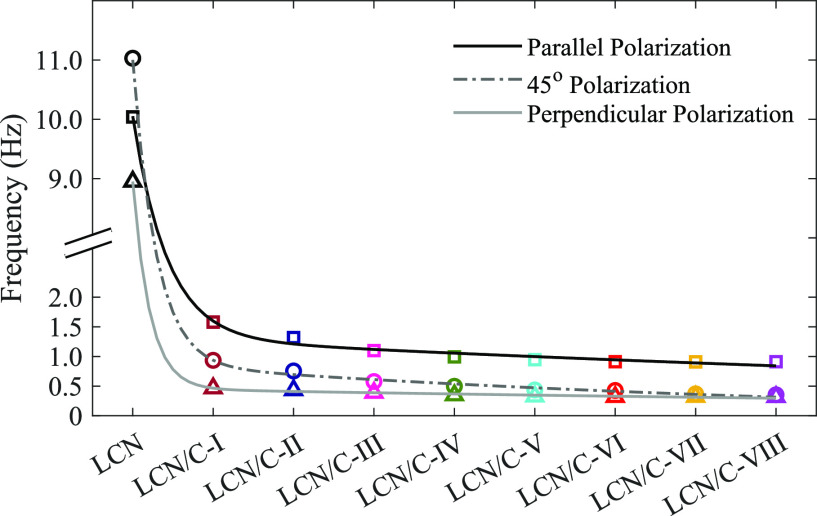
Oscillation frequency of all LCN films during irradiation with polarized light.

As can be seen, the oscillatory behavior of the LCN polymer is directly influenced by TiO_2_ nanoparticles. The amplitude and frequency of the oscillations of the LCN polymer are significantly reduced by the presence of a TiO_2_ dopant. Thus, in all oscillations, the highest and lowest oscillation frequencies are obtained when all LCN films are exposed to light with parallel and perpendicular polarizations, respectively (see Figure 8). In this regard, it is determined that, along with the reduction of oscillation characteristics in the presence of TiO_2_ nanoparticles, the oscillation frequency reduces along with the increase in the weight percentage of nanoparticles by comparing the findings obtained for the TiO_2_-doped films. For better analysis, the frequency rate of all resultant oscillations (*f*_⊥_/*f*_∥_) was calculated, and the values are listed in Table 3. The *f*_⊥_/*f*_∥_ values show a considerable decrease in the corresponding ratio when the LCN polymer is doped with TiO_2_ nanoparticles. This result shows a high difference between oscillation frequency values with perpendicular and parallel-polarized radiant light in the non-doped LCN film. Additionally, in the TiO_2_-doped LCN films, increasing the corresponding ratio is obtained simultaneously with the increase in the weight percentage of nanoparticles. Thus, LCN/C-I and LCN/C-VIII have the lowest and highest *f*_⊥_/*f*_∥_ values, respectively.

However, to produce the most accurate and correct analyses relating to the oscillatory behavior of the LCN polymer, looking into the influencing aspects is highly needed. Following the extensive previous studies, it has been determined that the oscillation frequency depends on the influencing factors as shown in the following equation:^41^
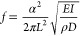
1

The values of α, *L*, *I*, and *D*, which express the oscillation mode constant, cantilever length, moment of inertia, and area of the cross-section, respectively, in all LCN films, are the same because their oscillation mode and dimensions are the same. Thus, the variable factors in the present study are the elastic modulus (*E*) and the density (ρ) of the films. By knowing the values of all the given variables, it is possible to determine the elastic modulus of the LCN polymer. For this reason, the elastic modulus of all LCN films was determined by substituting the values of all the components mentioned earlier with their density and oscillation frequency provided in Tables 2 and 3, respectively. It should be emphasized that, by taking into consideration the oscillation frequencies with 45° polarized radiant light, these calculations were made. Because according to Malus’s law,^42^ all oriented layers in the polymer bulk contribute to the absorption of radiation light with the mentioned polarization. The results of the calculations are provided in Table 4, which demonstrates the impact of utilized nanoparticles in reducing the elastic modulus of the LCN polymer. Therefore, the elastic modulus of TiO_2_-doped LCN films is substantially lower than the non-doped film. This is why the elastic modulus of TiO_2_-doped LCN films decreases as the weight percentage of the nanoparticles increases. Thus, the highest and lowest elastic modulus of TiO_2_-doped LCN films belongs to LCN/C-I and LCN/C-VIII, respectively. The elastic modulus of all LCN films persuades us to experimentally investigate the execution of additional investigation. Particularly, the relevant measurements were carried out at a certain temperature similar to the hinge point of the films during oscillation (approximately 57 °C). As listed in Table 4, the values obtained using the two indicated methodologies show the same changing trend with the measured values being greater than the calculated ones. The discrepancy in the measured values is caused by the non-uniformity of the stress imposed on the LCN films under the experimental measurement conditions of applying homogeneous heat and the heat created as a result of the photothermal effect when the films are exposed to light. In this approach, subjecting the film to the light source’s radiation puts it under more stress. It is important to note that the elastic modulus of the LCN polymer increases in the case of obtaining a chemical bond between the utilized nanoparticles and the polymer network or strengthening the inter-chain interaction in the polymer matrix.^43^ The result shows that, in the TiO_2_-doped LCN, the effective interaction between the LCN polymer chains decreases. It makes sense to obtain a lowering trend in the TiO_2_-doped LCNs’ elastic modulus given the absence of a new band in the FT-IR spectrum. This supports the weakened inter-chain interactions in the LCN polymer matrix in the presence of TiO_2_ nanoparticles. As a result, variations in the elastic modulus of the LCN films lead to the previously described variations in oscillation frequencies.

**Table 4:** Elastic Modulus (MPa) Derived from Two Methodologies: Experimental and Equational

polymer film	calculated elastic modulus(MPa)	measured elastic modulus(MPa)
LCN	406	605
LCN/C-I	4.05	5.35
LCN/C-II	2.65	3.46
LCN/C-III	1.56	2.13
LCN/C-IV	1.16	1.69
LCN/C-V	0.90	1.39
LCN/C-VI	0.87	1.21
LCN/C-VII	0.66	0.98
LCN/C-VIII	0.61	0.91

**Figure 9:**
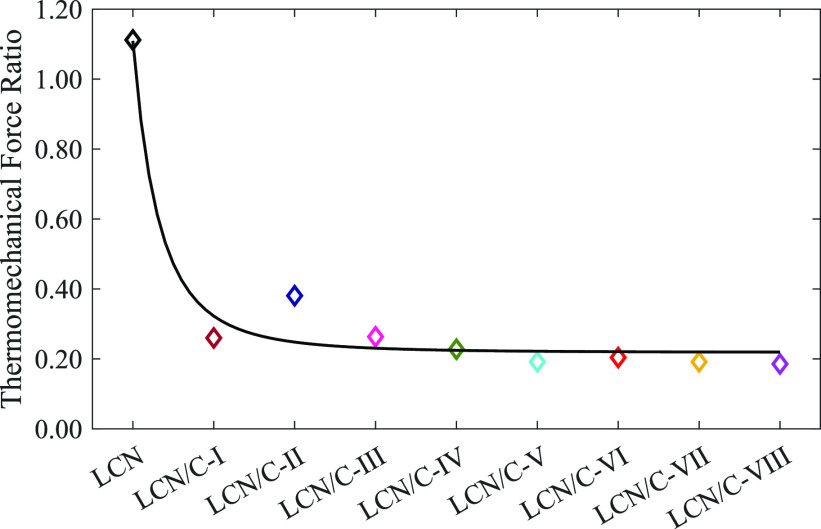
Thermomechanical force rate (*F*_⊥_/*F*_∥_) of all LCN films.

In addition to the findings mentioned above, it is crucial to consider how nanoparticles affect the thermomechanical force to achieve oscillatory behavior. Because of the contraction of the planar alignment surface and the expansion of the homeotropic alignment surface, this force causes an oscillating motion in the cantilever by generating torque. The relevant force can be calculated through the following equation:^44^
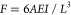
2where *A*, *E*, *I*, and *L* are the oscillation amplitude, elastic modulus, a moment of inertia, and the length of the studied films, respectively. It is noteworthy that, for this purpose, the values of the calculated elastic modulus were used because it is important to be influenced by the experimental conditions to achieve the oscillating movement. Due to the distinct alignment on each surface of LCNs, the different impacts are predictable for light polarization on their oscillatory behavior. To make a more accurate comparison possible, the thermomechanical force rate (*F*_⊥_/*F*_∥_) was calculated. The results of these calculations, which are drawn in Figure 9, indicate a significant reduction in the related rate when the LCN polymer was doped with TiO_2_ nanoparticles. Additionally, in TiO_2_-doped LCN films, the corresponding rate falls as the weight percentage of nanoparticles rises. This, in turn, illustrates the impact of TiO_2_ nanoparticles on the contraction and expansion of the polymer layers and surfaces in response to light exposure.^45^

**Figure 10:**
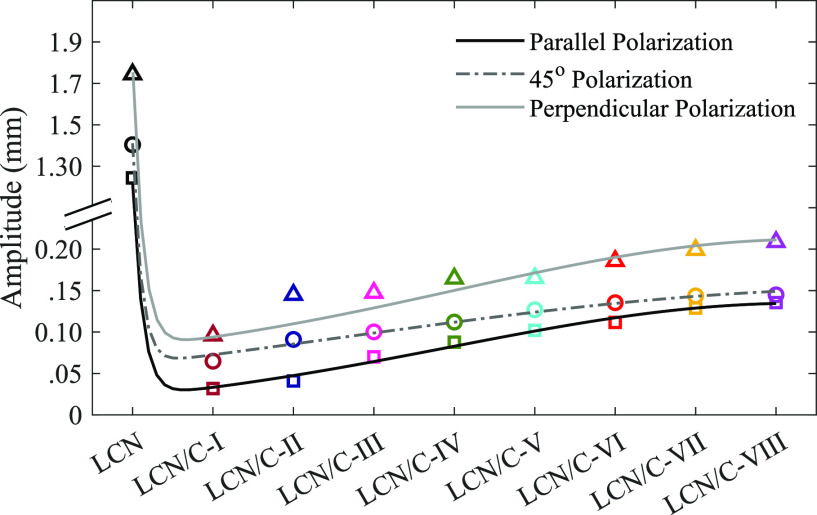
Oscillation amplitude of LCN films with irradiation of polarized light.

The effectiveness of the oscillation amplitude in the presence of TiO_2_ nanoparticles is shown in Table 3, where a considerable reduction in the oscillation amplitude of the LCN polymer in the presence of TiO_2_ nanoparticles has been observed. As it is known, doping the LCN polymer with TiO_2_ nanoparticles causes a disturbance in the orientation of LCN chains. Thus, it affects the contraction and expansion of the LCN polymer bulk and surface. The reduction of the related oscillation amplitude in the TiO_2_-doped LCN films with the increase in the nanoparticle’s weight percentage was observed. In such conditions, the nanoparticles increase the ordering of polymer chains and the amplitude. These findings and the surface roughness rates are shown in Table 2. This indicates the influence of TiO_2_ nanoparticles on the LCN polymer’s available surface for bending, which directly affects the oscillation amplitude. The high surface roughness ratio is the expression of the more available surface, which subsequently leads to an increase in the oscillation amplitude. As shown in Figure 10, the resultant oscillation amplitude in all polarized light increases in TiO_2_-doped LCN films with the increase in the weight percentage of the dopant.

**Figure 11:**
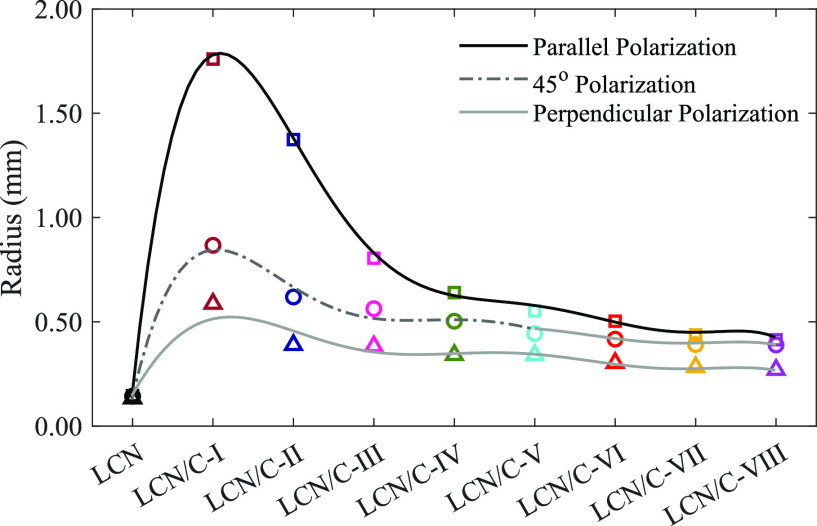
Curvature radius of all LCN films.

It is essential to note that a hypothetical circle is formed around the polymerized films due to the change of their shape from flat to a curve during oscillation. To analyze the impact of the nanoparticles on the created curvature, the change in the radius of these hypothetical circles was investigated. The curvature radius (*R*) can be carried out through eq 3:^44^
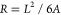
3where *L* and *A* are the length of the studied films and the oscillation amplitude, respectively. The obtained results are depicted in Figure 11, which indicates a rise in the corresponding radius when the LCN polymer is doped with TiO_2_ nanoparticles. In addition, with the increase in the weight percentage of nanoparticles, there is a decreasing trend in the TiO_2_-doped LCN films. As written in eq 3, the oscillation amplitudes impact the related radius values. Because the lowest amplitude belongs to LCN/C-I, its radius is more than that of all LCN films. Therefore, the hypothetical circle shrinks as the oscillation amplitude increases along with the increase of associated curvature.

## 5 Conclusions

In this work, a light-powered actuator was fabricated using UV-light absorbers. To fabricate a light-powered LCN polymer actuator, we used TiO_2_ nanoparticles as an inorganic UV-light absorber due to their great chemical stability and low toxicity. Investigations into the effect of TiO_2_ nanoparticles on the LCN polymer’s oscillation behavior were done after establishing the nanoparticles’ neutrality, the lack of impact on the splay molecular orientation in the polymer bulk, and the homogeneous dispersion in the polymer matrix. A significant decrease in the LCN polymer’s elastic modulus was observed by determining the elastic modulus of all LCN films. Our results showed that TiO_2_ nanoparticles with different weight percentages affect the LCN polymer’s oscillatory behavior where the oscillation characteristics of TiO_2_-doped LCN films were significantly less than the non-doped film. This reveals the strength of the influencing factors in this behavior. In such a way, by looking at thermomechanical force values, it turned out that TiO_2_ nanoparticles had an impact on polymer bulk and surface contraction and the expansion to produce oscillating motions. This is due to their existence altering the LCN inter-chain ordering, which was discovered by varying oscillation amplitudes. In addition, it was found that the curvature radius changed due to the presence of the TiO_2_ nanoparticles and the alteration of their weight percentage. Finally, it can be said that, in the applications of TiO_2_ nanoparticles where oscillation is necessary to prevent the polymer surfaces from being deactivated, the doped LCN with TiO_2_ nanoparticles can be used and can achieve the desired oscillation with the amount of doping. The LCN doped with TiO_2_ nanoparticles can be used for soft robotic systems from hardware for power and control.

## Supporting Information Available

Details of the results obtained from UV–vis spectra, EDX mapping, AFM images, and light-induced self-oscillation studies (PDF)
