# Supplementary material for: Light-Powered Liquid Crystal Polymer Network Actuator Using TiO2 Nanoparticles as an Inorganic Ultraviolet-Light Absorber
Source: ACS Omega. 2023 Mar 10;8(11):10555–64. doi: 10.1021/acsomega.3c00417 (PMC10034986; doi:10.1021/acsomega.3c00417)
Supplement: Supplementary file 1 — ao3c00417_si_001.pdf [file ao3c00417_si_001.pdf]

Supporting Information

Light-powered liquid crystal polymer network actuator using TiO_2 nanoparticle as an inorganic UV-light absorber

Zhila Alipanah[a], Mohammad Sadegh Zakerhamidi[a,b], Hossein Movla[a], Batool Azizi[c], Igor Muševič[d], Amid Ranjkesh[d,*]

[a] *Faculty of Physics, University of Tabriz, Tabriz, Iran*

[b] *Photonics Center of Excellence, University of Tabriz, Tabriz, Iran*

[c] *Central laboratory, University of Tabriz, Tabriz, Iran*

[d] *Condensed Matter Department, J. Stefan Institute, Jamova 39, Ljubljana, Slovenia*

* Corresponding author's E-mail address: amidranjkesh@gmail.com

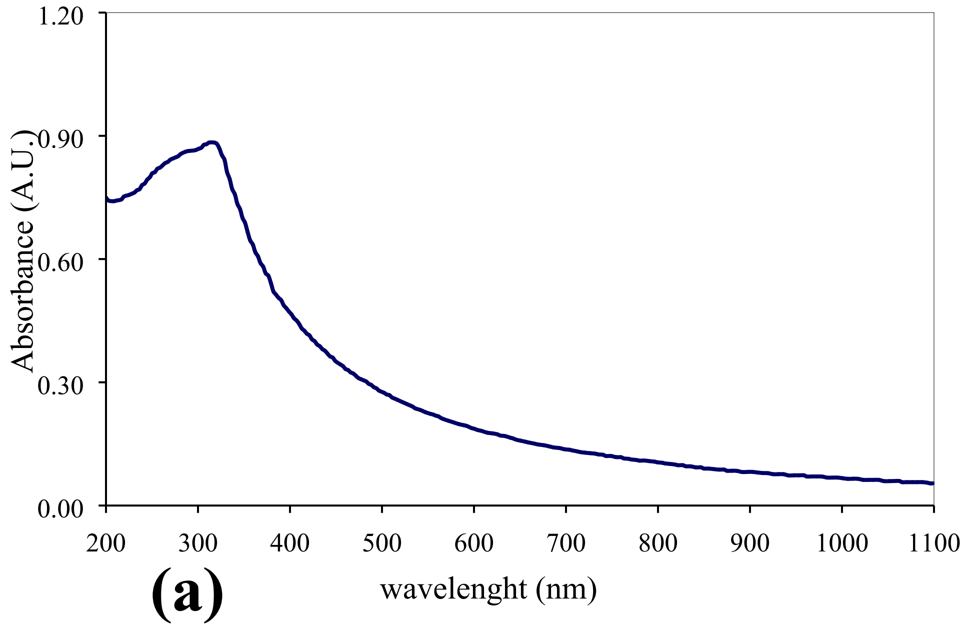

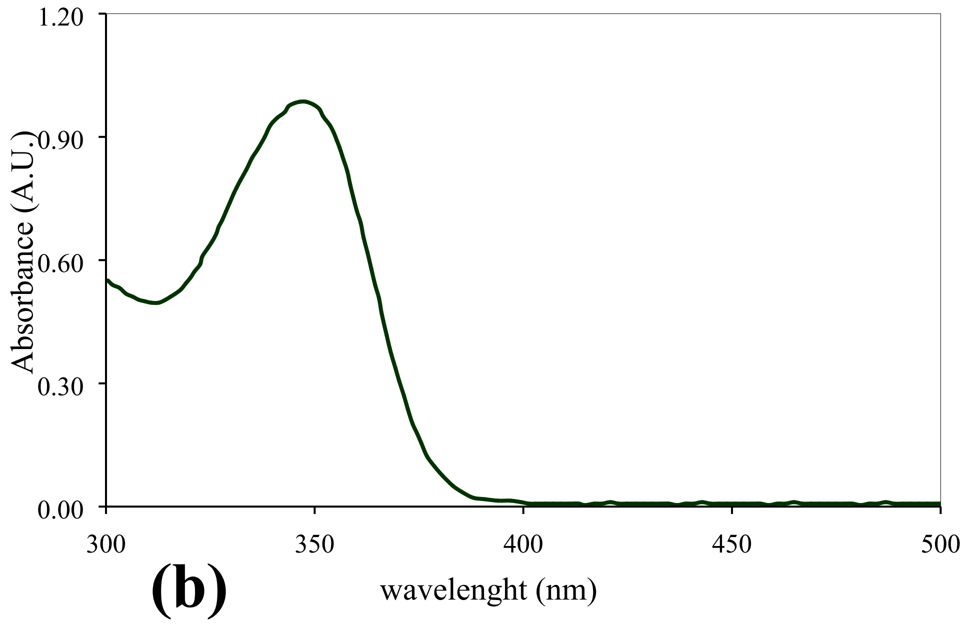

Figure. S1: UV-vis absorption spectrum using: a) TiO_2, b) Tinuvin460.

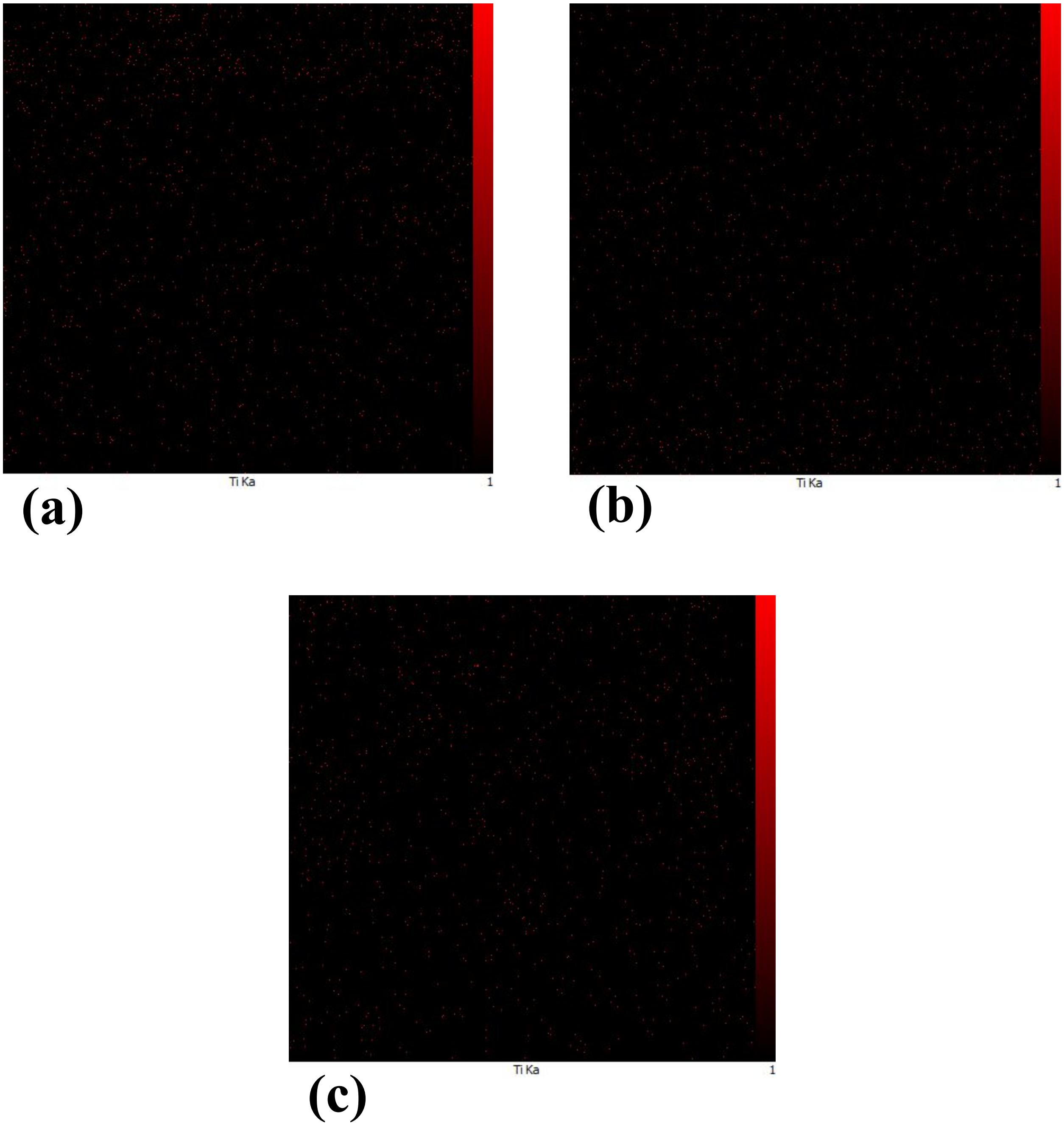

Figure. S2: EDX-map from the dispersion of Ti in the number of polymerized films: a) LCN/C-V, b) LCN/C-VI, c) LCN/C-VII.

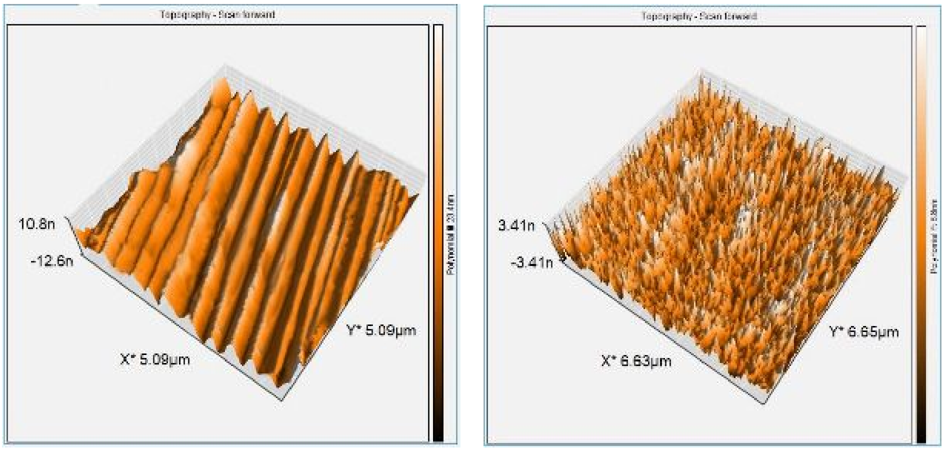
Topography - Scan forward
10.8n
-12.6n
X* 5.09µm
Y* 5.09µm
Topography - Scan forward
3.41n
-3.41n
X* 6.63µm
Y* 6.65µm

(a)

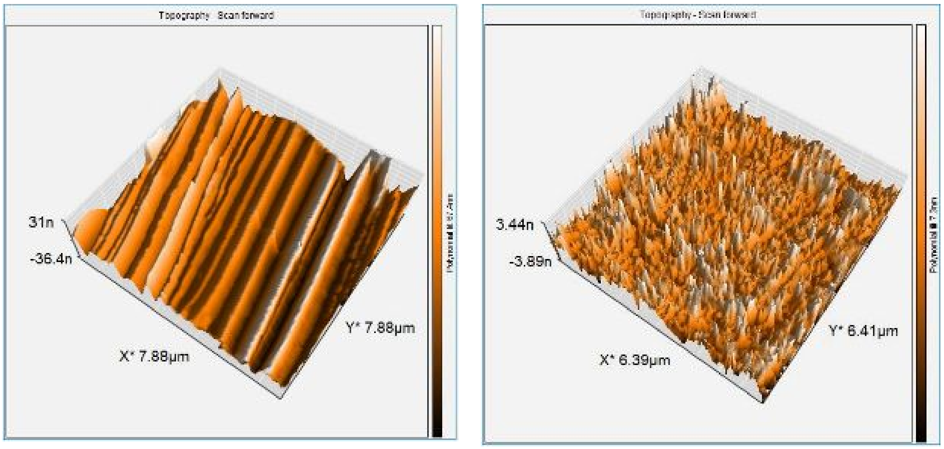
Topography - Scan forward
31n
-36.4n
X* 7.88µm
Y* 7.88µm
Topography - Scan forward
3.44n
-3.89n
X* 6.39µm
Y* 6.41µm

(b)

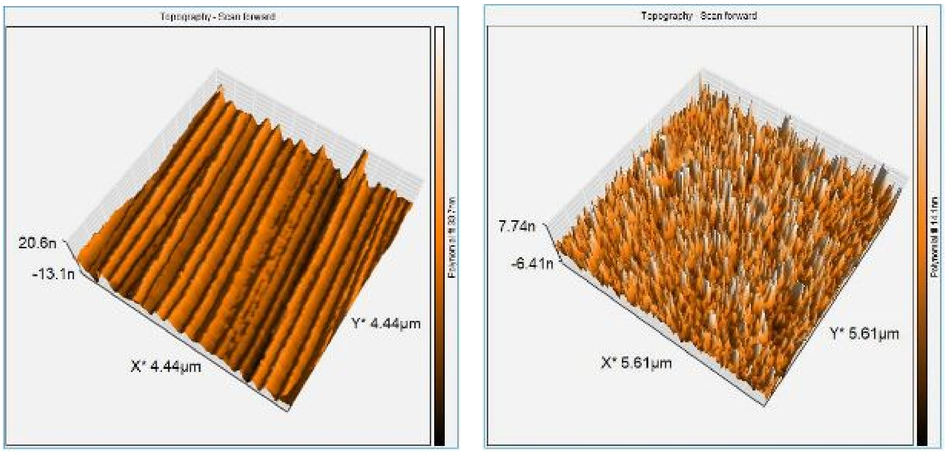
Topography - Scan forward
20.6n
-13.1n
X* 4.44µm
Y* 4.44µm
Topography - Scan forward
7.74n
-6.41n
X* 5.61µm
Y* 5.61µm

(c)

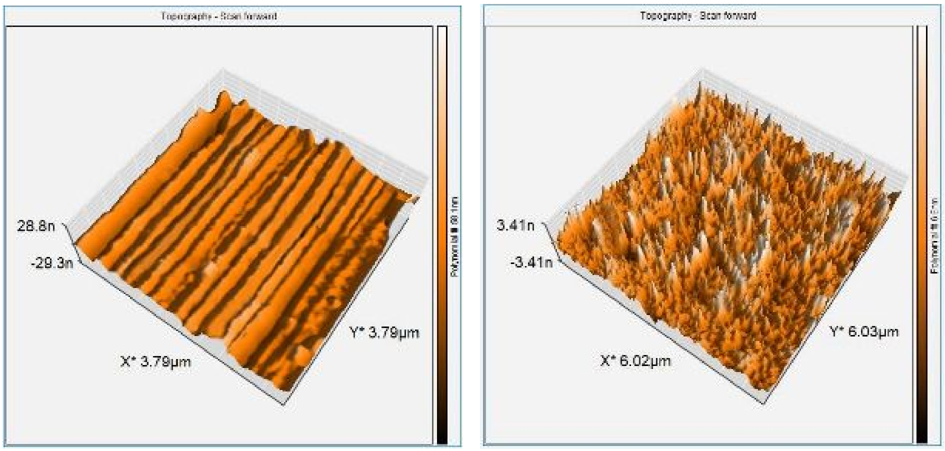
Topography - Scan forward
28.8n
-29.3n
X* 3.79µm
Y* 3.79µm
Topography - Scan forward
3.41n
-3.41n
X* 6.02µm
Y* 6.03µm

(d)

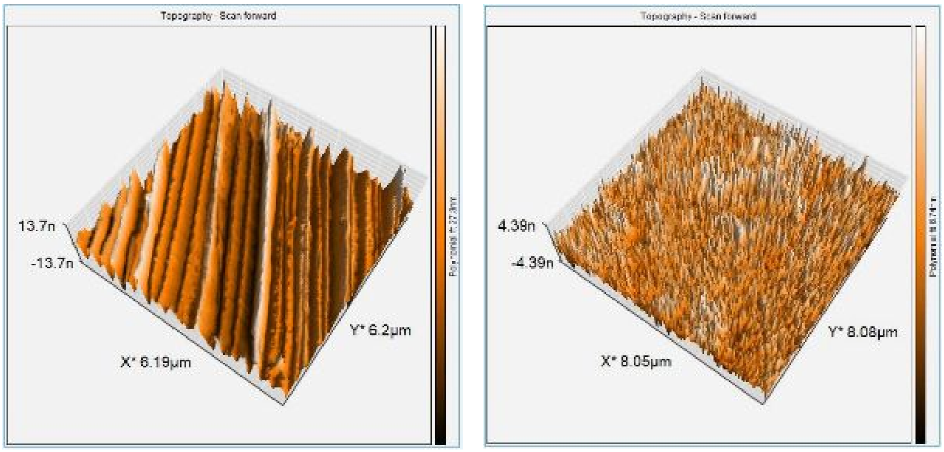
Topography - Scan forward
13.7n
-13.7n
X* 6.19µm
Y* 6.2µm
Topography - Scan forward
4.39n
-4.39n
X* 8.05µm
Y* 8.08µm

(e)

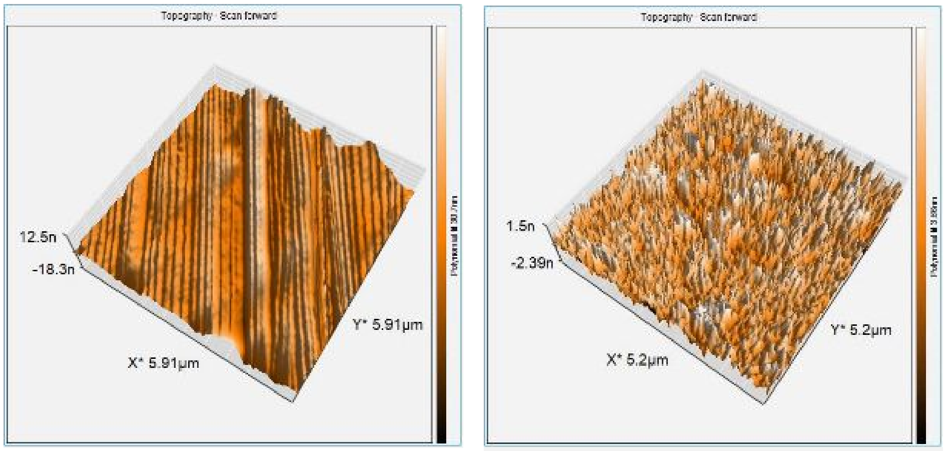
Topography - Scan forward
12.5n
-18.3n
X* 5.91µm
Y* 5.91µm
Topography - Scan forward
1.5n
-2.39n
X* 5.2µm
Y* 5.2µm

(f)

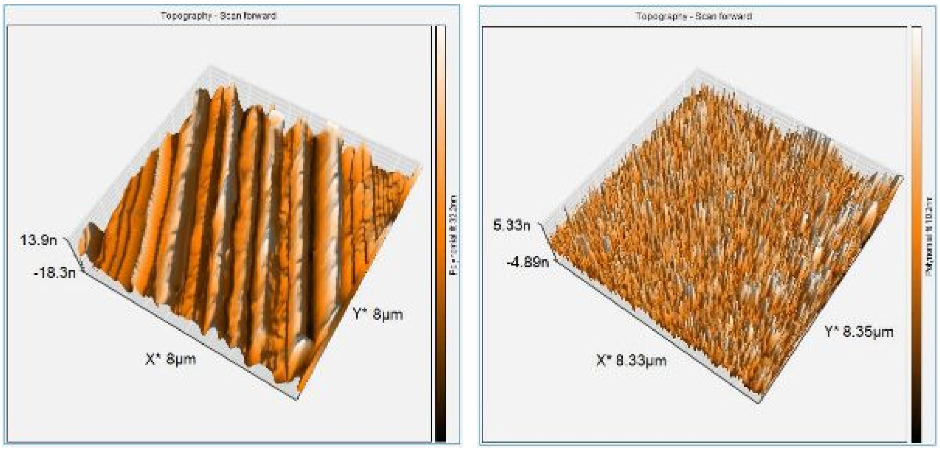

(g)

Figure. S3: AFM images of planar (images on the left) and homeotropic (images on the right) oriented surfaces: a) LCN/C-I, b) LCN/C-II, c) LCN/C-III, d) LCN/C-IV, e) LCN/C-V, f) LCN/C-VI, g) LCN/C-VII.

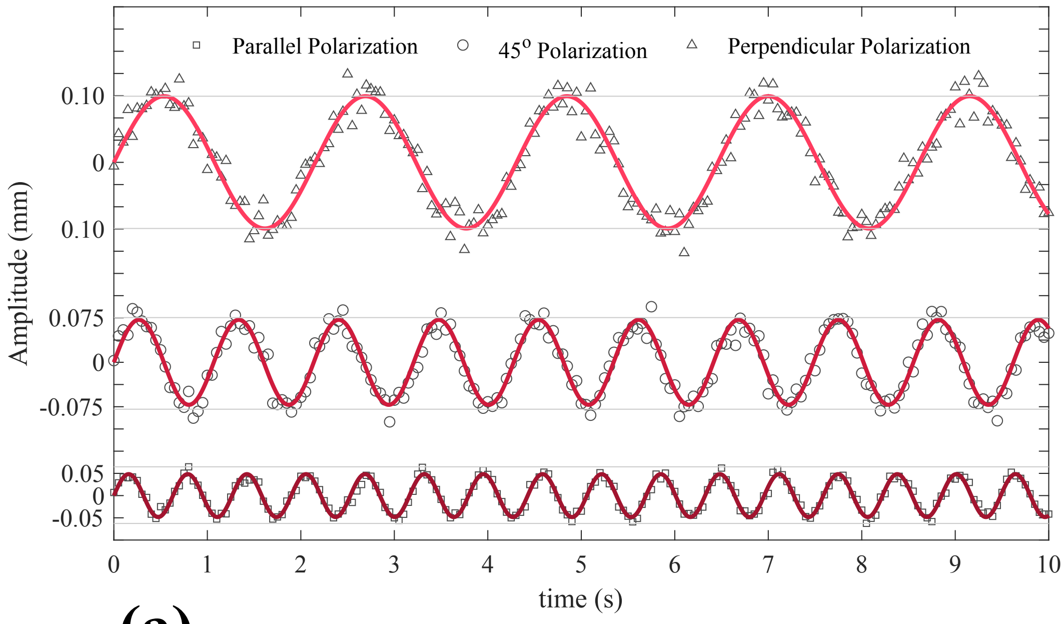

Parallel Polarization
45° Polarization
Perpendicular Polarization
0.10
0
0.10
0.075
0
-0.075
0.05
0
-0.05
Amplitude (mm)
0
1
2
3
4
5
6
7
8
9
10
time (s)

(a)

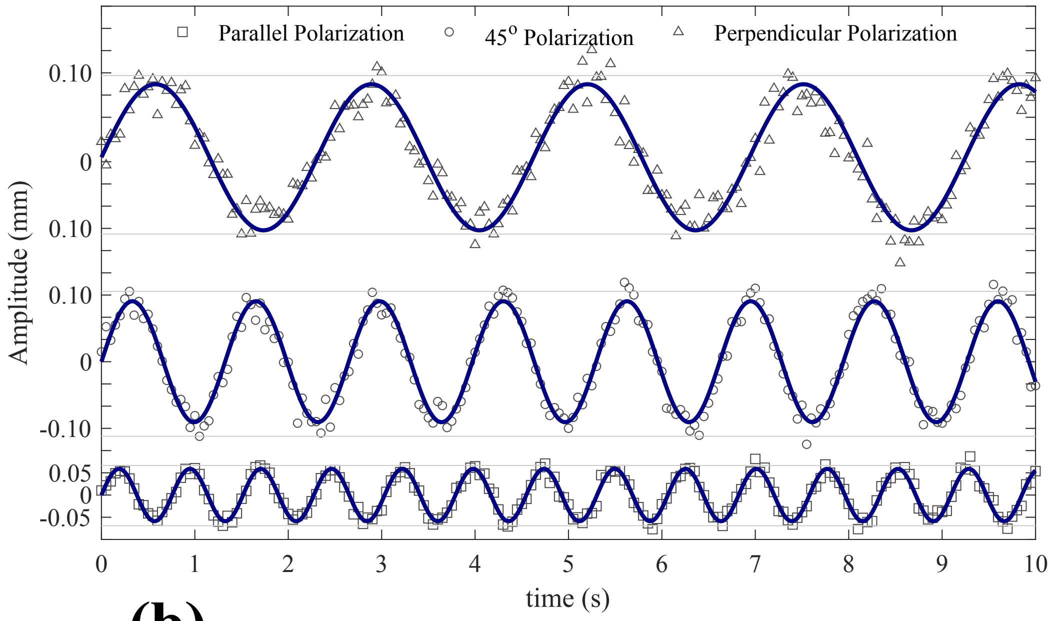

Parallel Polarization
45° Polarization
Perpendicular Polarization
0.10
0
0.10
0.10
0
-0.10
0.05
0
-0.05
Amplitude (mm)
0
1
2
3
4
5
6
7
8
9
10
time (s)

(b)

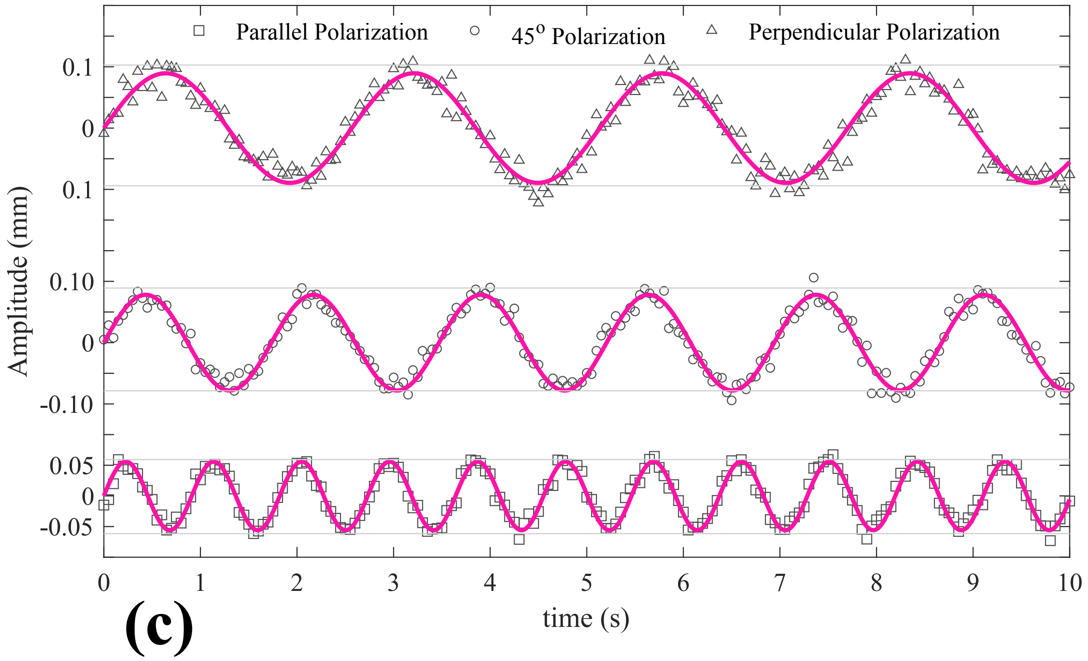
Parallel Polarization
45° Polarization
Perpendicular Polarization
0.1
0
0.1
0.10
0
-0.10
0.05
0
-0.05
Amplitude (mm)
0
1
2
3
4
5
6
7
8
9
10
time (s)

(c)

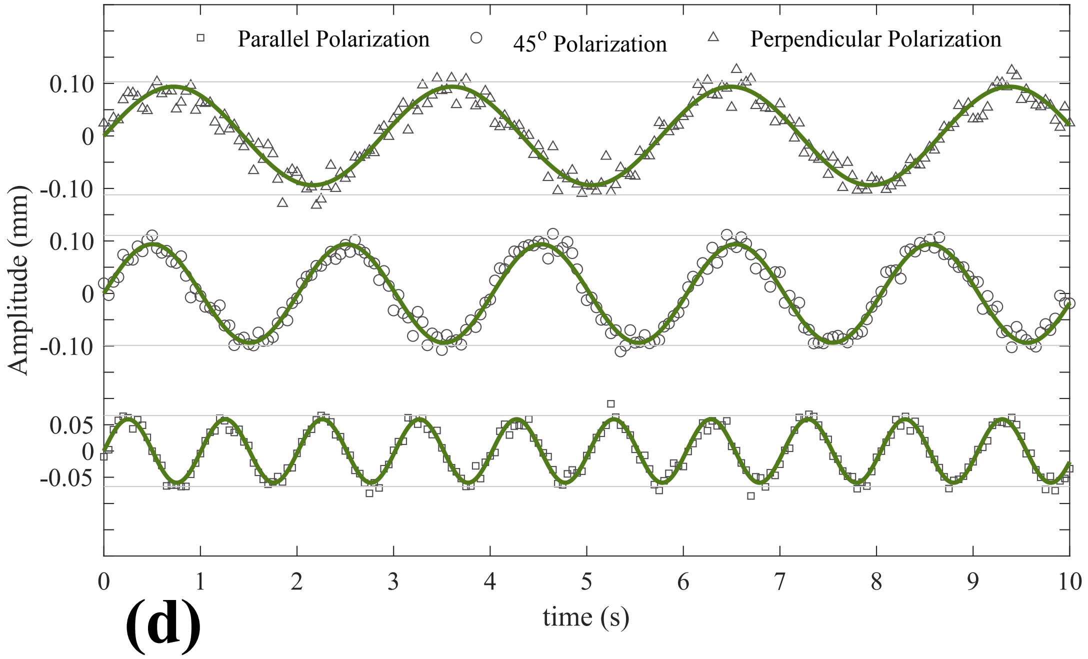
Parallel Polarization
45° Polarization
Perpendicular Polarization
0.10
0
-0.10
0.10
0
-0.10
0.05
0
-0.05
Amplitude (mm)
0
1
2
3
4
5
6
7
8
9
10
time (s)

(d)

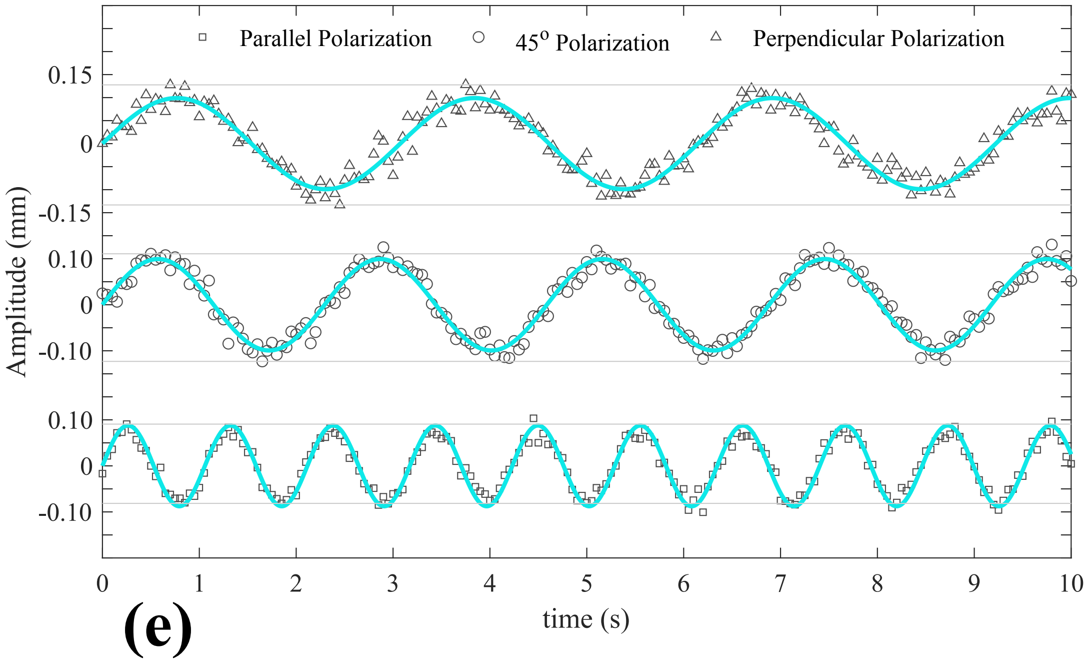

Parallel Polarization
45° Polarization
Perpendicular Polarization
Amplitude (mm)
0.15
0
-0.15
0.10
0
-0.10
0.10
0
-0.10
0
1
2
3
4
5
6
7
8
9
10
time (s)

(e)

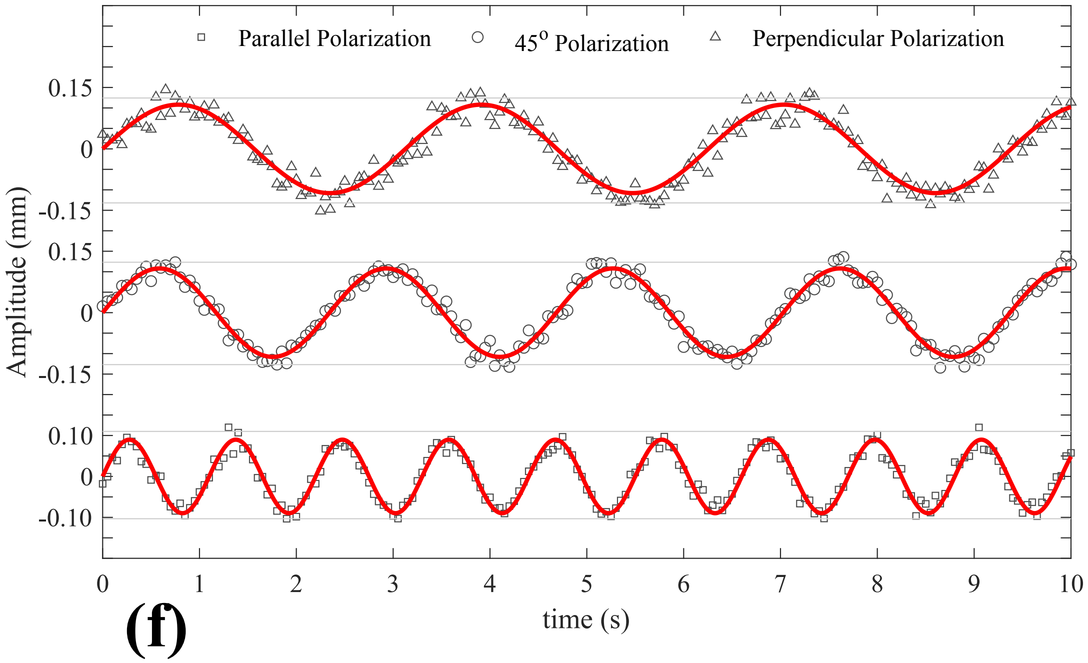

Parallel Polarization
45° Polarization
Perpendicular Polarization
Amplitude (mm)
0.15
0
-0.15
0.15
0
-0.15
0.10
0
-0.10
0
1
2
3
4
5
6
7
8
9
10
time (s)

(f)

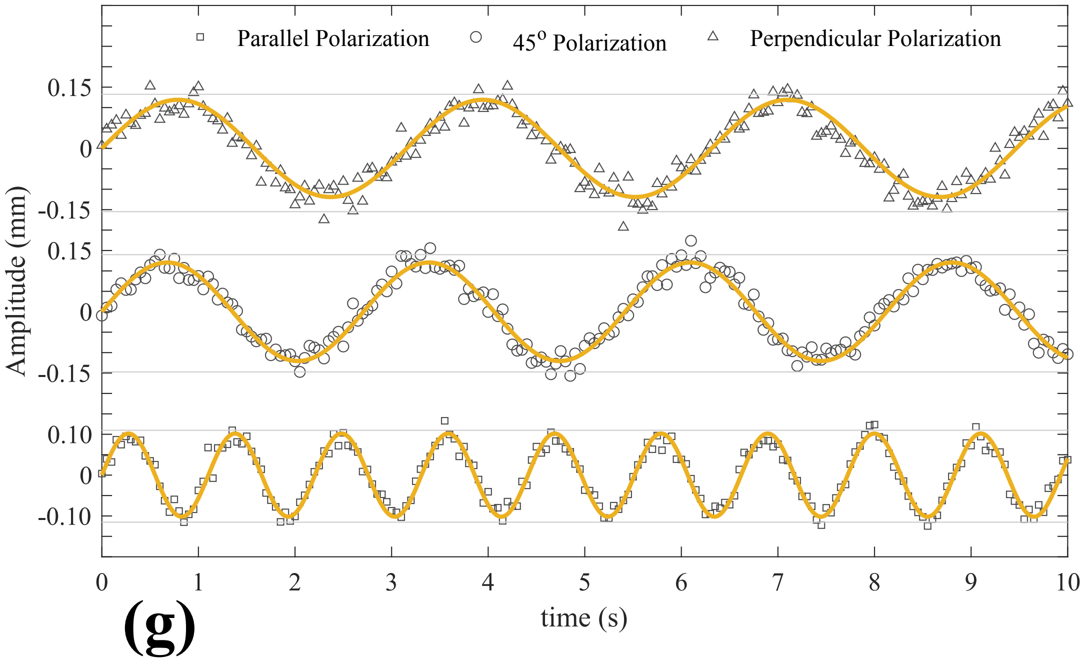

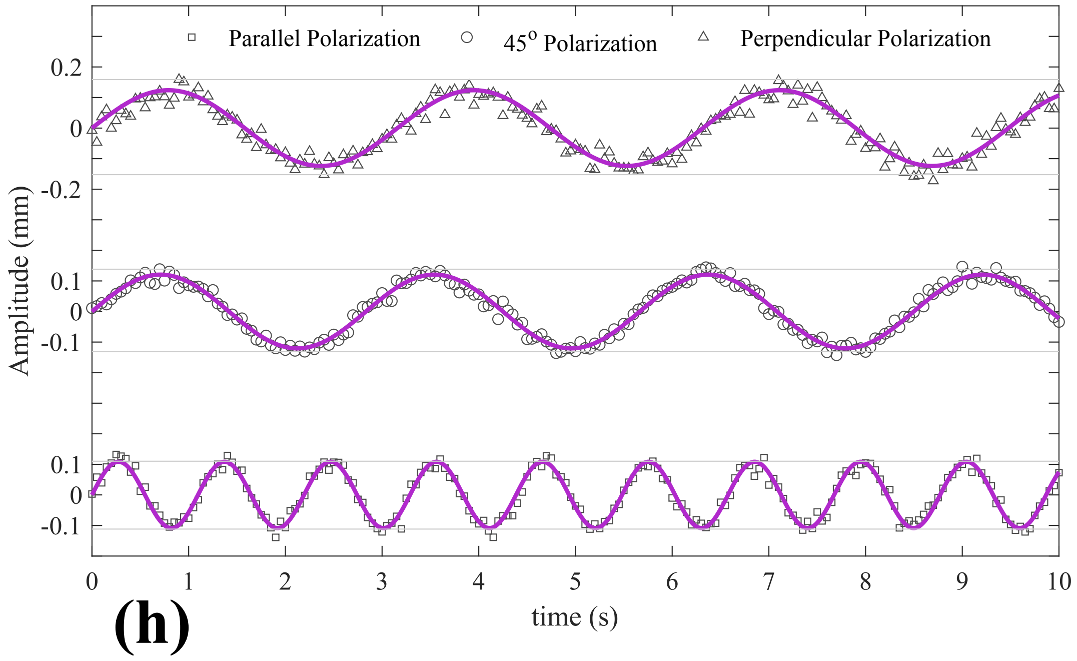

Figure. S4. Self-oscillation patterns during irradiation with polarized light: a) LCN/C-I, b) LCN/C-II, c) LCN/C-III, d) LCN/C-IV, e) LCN/C-V, f) LCN/C-VI, g) LCN/C-VII, h) LCN/C-VIII.
